# Hearing healthcare gaps in LMICS: snapshot from a semi-urban community in Nigeria

**DOI:** 10.4314/ahs.v21i2.53

**Published:** 2021-06

**Authors:** Adebolajo Adeyemo, Segun Ogunkeyede, Oluyinka Dania

**Affiliations:** 1 University of Ibadan, Institute of Child Health; 2 University College Hospital, Department of Otorhinolaryngology; 3 University of Ibadan, Department of Otorhinolaryngology; 4 University College Hospital, Department of Community Medicine

**Keywords:** Hearing loss, healthcare delivery, disease burden, ear diseases, developing countries

## Abstract

**Background:**

Low and middle-income countries (LMICs) have high prevalence of hearing loss which are mainly due to preventable causes. While urban communities in LMICs are likely to have functional hearing healthcare delivery infrastructure, rural and semi-urban communities may have different reality.

**Objectives:**

This study aimed to provide: (i) a snapshot of the burden of ear diseases and (ii) a description of available hearing healthcare resources in a semi-urban Nigerian community.

**Methods:**

A cross-sectional study of households selected by multistage random sampling technique. Seventy-four participants: 39 males and 35 females with mean age of 34 years ± 5.24 were recruited and answered a structured questionnaire. In addition, the availability of hearing healthcare services in 15 health centers within the community were determined.

**Results:**

All participants reported recent occurrence of ear complaints or gave similar history in a household member. Common complaints were ear discharge, ear pain and hearing loss. Medical intervention was sought from patent medicine stores, hospitals and traditional healers. None of the assessed hospitals within the study site was manned by an ENT surgeon or ENT trained nurse.

**Conclusion:**

Despite the heavy burden of ear complaints there is inadequate hearing healthcare delivery in a typical LMIC community. This highlights the need for urgent improvement of hearing healthcare.

## Introduction

The inability to hear adequately affects people of all ages with varying degrees on impact on the affected individuals. Multiple aspects of human social interaction are related to adequate hearing function; thus, loss of hearing ability exerts a debilitating effect on the psychology, quality of life, social relationships and motor skills of affected individuals^1^. Hearing loss (HL) in children results in diminished language development and educational progress^2^, reduction in quality of life is also seen in children with HL^3^. Among adults, HL causes occupational difficulties^2^ as well as cognitive impairment^4^. Age related hearing loss (ARHL) is associated with dementia and brisk deterioration of cognitive abilities^1^. Apart from cognitive difficulties, individuals with HL also experience social difficulties, thus, in all age groups there is often stigmatization of people with hearing loss^2^,^5^. Moreover, people with HL may experience reduced opportunities for progress, they are less likely to attend tertiary academic institutions compared to individuals with normal hearing, they endure doubling of the stress at work with reduced labour participation compared to people with normal hearing profile^6^. However, these huge impact of HL on individuals, families and communities is often underestimated due to the ‘hidden’ nature of HL^5^. A delay in the early identification, diagnosis and appropriate management could cause worsening of hearing loss^7^ and may significantly exacerbate the impact of the disability on affected individuals. There is therefore a growing need for adequate screening, identification and intervention services for hearing loss worldwide but more importantly in developing countries^8^.

Low and middle-income countries (LMICs) such as Nigeria and many Sub-Saharan African countries have about 80% of world's deaf and hearing-impaired population^9^. The majority of hearing disorders in LMICs often go untreated resulting in life-long hearing impairment^10^. Moreover, in LMICs, attention and prioritization of resources is often diverted to preventable life-threatening illnesses such as malaria. This may have worsened the provision of ear healthcare in LMICs as hearing loss is not considered the highest health priority during disbursement of resources 10. Despite the limited resources in LMICs, proper deployment of primary health care services can provide the most effective interventions for hearing disorders 11. Primary health care services ought to cover ear care under its management umbrella of common ailments, especially given that ear problems are amongst the commonest ailments in infants and children^12^.

Unfortunately, the general lack of attention to ear care at global and national levels^13^ is a significant contributor to the global burden of disease. The inattention to ear care is pronounced in LMICs where the primary health care facilities are ill-equipped in manpower and material resources to provide ear health care,^14^ thereby resulting in a high burden of hearing disability in these countries. The huge disease burden as a result of inadequate ear health is largely preventable since half of all cases of hearing loss could be avoided if there is effective primary prevention 15. The rapidly growing population in LMICs without a proportionate growth in resources to manage ear disorders implies a likely increase in the number of people with hearing disabilities^16^,^17^. This study aims to provide a snapshot of the burden of ear diseases and hearing health care resources in a semi-urban Nigerian community. The implication is the provision of scientific evidence on the need for appropriate ear care intervention and advocacy efforts for adequate resources allocation.

## Methods

This is a cross-sectional community-based study carried out in Oyo town in Southwestern Nigeria among households and health facilities. Oyo town is made up of 3 local governments areas (Oyo East, Oyo West and Atiba local government areas) with 30 electoral wards and a population of 428,798 with male to female ratio of 1:1.75 18. The majority of the inhabitants are peasant farmers; the others are either self-employed in a trade, government employees or work in some of the small-scale industries in the town. Yoruba and English languages are the predominant languages spoken in the town. Oyo town is served by 68 primary health clinics, one general hospital (secondary level health facility) and several private hospitals manned by general practitioners. There are no tertiary level health facilities in the town.

Ethical approval was obtained from the Ethics Committee of the Oyo State Ministry of Health, Nigeria. Written permission from community leaders and informed consent from each participant was also obtained. Confidentiality was preserved by not collecting personal identifiable information from the participants. A multistage random sampling technique was used to select the participants from the 30 electoral wards in the community. Briefly explained: All the electoral wards were roughly similar in size. Fifty percent of the electoral wards (n=15) were randomly selected. A convenient sample size of 5 housing units per eletoral ward was made. These housing units were chosen in a systematic random manner from the selected electoral wards. Prior to the commencement of the study, the housing units in each electoral ward had been numbered serially by a government agency – the National Population Commission - the housing unit corresponding to the first serial number was selected and subsequent units were selected at interval of five (5) houses apart. When occupants of a selected unit were unavailable or declined to participate in the study the next housing unit was selected as replacement.

A structured questionnaire (see appendix 1) was administered to study participants. The questionnaire was translated into Yoruba via a back-translation method 19. This involved (a) direct translation from English to Yoruba by speakers fluent in Yoruba and English, (b) re-translation of the Yoruba questionnaire by an independent party of speakers fluent in both languages and (c) comparison of the original English version and the re-translated version of the questionnaire by ENT surgeons fluent in both languages, areas of disagreements were resolved to ensure accuracy of the tool. An adult ≥18 years from each of the chosen households was randomly selected for the interview; a total of 74 individuals were recruited into the study (Table 1). After obtaining written informed consent, the questionnaires were administered. The participants who were not fluent in English were administered the Yoruba version of the questionnaire. The questionnaires were administered in direct interviews, which took place in the participant's home. One health facility (primary health clinic) was randomly selected in each of the chosen 15 electoral wards. Demographic data and clinical variables were presented using percentages, tables and charts as appropriate while summary statistics were done using means and proportions. A reliability co-efficient of 0.823 using the Cronbach's Alpha model was obtained for the questionnaire used in this study, this showed good reliability of the tool in achieving the goals of the study.

**Table 1:** Socio-demographic data of participants (n=74)

Variable	Frequency	Percentage
**Age (years)**		
**17–24**	12	16.2
**25–34**	29	39.2
**35–44**	15	20.3
**45–54**	6	8.1
**55–64**	7	9.5
**65–74**	3	4.0
**75–84**	2	2.7
**Total**	74	100.0
**Sex**		
**Male**	39	52.7
**Female**	35	47.3
**Total**	74	100
**Monthly Income in Naira**		
**₦0 – ₦5,000**	56	75.6
**₦6,000 – ₦10,000**	8	11.0.
**₦11,000 – ₦15,000**	5	6.7
**₦16,000 – ₦20,000**	5	6.7
**Total**	74	100.0

### Sociodemographic characteristics of respondents

The majority (59.5%) of the participants were within 25 to 44 years of age (mean age = 34 ±5.2 years) and married in a monogamous family setting. There was an almost equal sex and religion distribution. Most of the participants earn a monthly income >5000Naira (14 US Dollars) {1USD = 360 NGN} (Table 1).

## Results

The results were sequentially presented, starting with burden of ear diseases and then available health care resources.

### Burden of Ear Diseases

#### Common Ear Problems in the Community

All the participants reported a history of ear complaints or in a member of their household, while 74.3% (n=55) reported ear complaints within 9 months preceding the study. The most commonly reported ear complaint is ear discharge (87.5%) and ear pain (72.9%). (Table 2).

**Table 2:** Reported Ear Problems (n=55)

Types of Ear Problem	Frequency
Ear pain	40 (72.7%)
Ear discharge	48 (87.3%)
Ringing sensation	24 (43. 6%)
Foreign body	19 (35.0%)
Trauma to the ear	11 (20.0%)
Hearing loss	26 (47.3%)
Feeling of fullness in the ear	14 (25.5%)
Wax impaction	23 (41.8%)
Ear itch	23(41.8%)

Some individuals had more than one symptom

### Source of Health Care Services

Participants with ear complaints sought medical intervention from chemist shops (28, 51.0%), hospital care (16, 29.0%) and traditional health providers (11, 20%). Amongst those who sought hospital care, (11, 69.0%) were treated in private general practice hospitals, (3, 19.0%) were treated in public general hopitals while (1, 6.0%) each were treated at primary health care centers and tertiary hospital. The cadres of health professionals who offered treatment within the hospital care category were: nurses (7, 43.7%) [primary health clinics and public general hospitals], general practitioners (8, 50.0%) [private general practice hospitals and public general hospitals] and ENT specialist (1, 6.3%) [tertiary hospital]. The ENT specialist care was provided in tertiary hospital located outside the study community.

### Ear Care Needs Assessment

All respondents reported a need for facility-based ear care service at the primary health clinics (PHCs), provision of visiting ear specialist as well as provision of nurses trained in ear care. See Table 3. Majority (49, 66.2%) reported willingness to pay an amount less than 2000 Naira (6 US Dollars). See Table 4.

**Table 3:** Ear Care and Human Resource Need Assessment (n=67)

Statements	Yes	No	Total
Do you think this community needs a provision of ear care services in its health facility?	67	0	67
Provision of a resident ear doctor.	59	1	60
Provision of a visiting ear doctor.	26	0	26
Provision of just any doctor.	9	6	15
Provision of a nurse trained in ear care.	50	0	50

**Table 4:** Willingness to Pay (n=74)

Amount (in Naira) {1USD = 360 NGN}	Number of respondents	Percentage
Free treatment	17	23.0
₦200	1	1.4
₦500	13	17.6
₦1,000	18	24.3
₦2,000	18	24.3
₦5,000	6	8.1
₦7,200	1	1.4
Total	74	100.0

### Assessing Gaps in Ear Care Services Provision in Available Health Services

This section provides results for the second aim of the study.

There were no trained medical personnel for ear care services in any of the 15 selected health care facilities within the study community. None of the facilities assessed had appropriate materials and equipment for ear services. Table 5

**Table 5:** E.N.T Equipment Availability in PHCs (n=15)

Equipment	Available
Cotton applicators, 14cm, serrated.	0 (0%)
Head light (spare bulbs).	0 (0%)
Head mirror.	0 (0%)
Ear syringe (metal) 50ml.	15 (100%)
Kidney bowls.	15 (100%)
Otoscope	5 (33%)
Disposable specula – 2.5mm and 4mm.	6(25%)
Syringes – plastic, various sizes.	13 (87.0%).
Equipment for doing simple maintenance on hearing aids and ear molds.	0 (0%)

## Discussion

Research on health as a critical component of national development has continued to receive adequate attention in literature due to the fact that investment in human capital through health could bring about economic growth and development 20. The present health care service delivery in Nigeria is hospital based and concentrated around urban centers, as a result people residing in rural areas often lack many basic needs including ear care services. This lack of ear care services in rural communities is not only seen in Nigeria as many other countries have similar imbalance in their health system^21^.

### Burden of Ear Diseases

#### Common Ear Problems in the Community

Ear discharge was the most common ear problem in the community, a situation which has been demonstrated by similar studies^22^,^23^. This may be due to preventable factors such as poor hygiene and malnutrition that predisposes to suppurative otitis media^24^. The high humidity seen in the tropical rainforest clime of southwest Nigeria could contribute to the high prevalence of ear discharge^25^; in addition, the irrational use of antibiotics within the community may also predisposes to high level of bacteria resistance and subsequent persistence of ear infection^22^,^23^. This is an important regulatory issue in many LMICs where individuals can purchase otherwise prescription-only drugs, on-demand over the counter. Earwax impaction is a major health problem worldwide affecting the general population and a main cause of health consultations^26^,^27^. The prevalence of wax impaction in the index community appears higher than what had been earlier reported in the general population^26^. The habitual ear cleaning that is often practiced may disturb the natural ear self-cleaning process of the cerumen and may cause excessive cerumen production via stimulating the cerumen gland^28^,^29^. This high burden of wax impaction may also reflect in common availability of instruments required for ear syringing in all the visited heath care units. The pattern of ear problems in this study serves as pointer to the level of development of the area and also emphasizes the need for preventive measures in solving the ear care challenges rather than curative measures.

### Source and Quality of Health Care Services

The quality of hearing healthcare care experienced by the study participants is doubtful as the majority of the participant's source for care from drug vendors at patent medicine shops. This defect in hearing healthcare delivery is a major source of concern in many LMICs. Since patent medicine vendors and the traditional care givers play a significant role in the healthcare delivery of rural dwellers^30^,^31^, it may be necessary to incorporate them into delivery of hearing healthcare at the primary health care level by providing them appropriate training using tools such as the World Health Organization (WHO) training manual^32^. This will improve the access to hearing healthcare that is available to rural community dwellers and prevent unnecessary complications^33^.

Since otolaryngology specialty care is largely lacking in the rural community, community members who sought care in private clinics and general hospitals were attended to by general practitioners. This pool of health care practitioners is another important cadre of personnel in delivering middle level hearing healthcare, especially in rural communities. Tapping this pool requires provision of continuous medical education in otolaryngology to update their knowledge and practices and improve their ability to provide care.

### Assessing Gaps in Ear Care Services Provision

All respondents reported lack of ear care service in the community and the need to provide facility-based ear care service at the primary health clinics. The lack of ear care services in rural communities is a common trend seen not only in Nigeria but in many LMICs too^34^–^37^. In Nigeria – in a model similar to many LMICs - health care service delivery is built around a model of hospitals based in urban centers^36^ thus, people residing in rural areas lack many basic needs including ear care services; as evidenced by none of the facilities assessed had adequate basic materials or equipment for ear care. The study respondents reported a need for facility-based ear care service, provision of visiting ear specialist and willingness to pay for ear care services, however, there were no otolaryngologist or trained ENT nurse in any of the health facilities visited. Despite the willingness of the community members to pay for specialist services, the lack of guaranteed funds to pay for care may limit the ability to commit a specialist to visit rural or semi-urban health facilities regularly. The introduction of community health insurance for rural dwellers could provide assurance of payment for services and thus encourage regular visits by specialists to rural hospitals with subsequent improvement in health outcomes^38^.

Ear diseases are a significant component of health demands in semi-urban and rural areas of LMICs, however, there is lack of commensurate trained health personnel to attend to these needs.

Multiple pronged actions are required to address the gaps in hearing healthcare delivery. These actions could include adoption of the Primary Ear Care recommendations of the WHO^32^ into service delivery at the primary health clinics and adoption of telemedicine in healthcare delivery in LMICs.
